# Copper-induced increased expression of genes involved in photosynthesis, carotenoid synthesis and C assimilation in the marine alga *Ulva compressa*

**DOI:** 10.1186/s12864-018-5226-4

**Published:** 2018-11-20

**Authors:** Felipe E. Rodríguez, Daniel Laporte, Alberto González, Katterinne N. Mendez, Eduardo Castro-Nallar, Claudio Meneses, Juan Pablo Huidobro-Toro, Alejandra Moenne

**Affiliations:** 1 Faculty of Chemistry and Biology, University of Santiago of Chile, Av. Libertador Bernardo O’Higgins, 3363 Santiago, Chile; 2 Center for Bioinformatics and Integrative Biology, Facultad de Ciencias de la Vida, Universidad Andrés Bello, Santiago, Chile; 3 Center of Plant Biotechnology, Facultad de Ciencias de la Vida, Universidad Andrés Bello, Santiago, Chile; 4 FONDAP Center for Genome Regulation, Universidad Andrés Bello, Santiago, Chile; 5 Center for the Development of Nanoscience and Nanotechnology (CEDENNA), Santiago, Chile

**Keywords:** Calvin cycle, Carotenoid level, Copper, Marine alga, Photosynthesis, *Ulva compressa*

## Abstract

**Background:**

The marine alga *Ulva compressa* is the dominant species in coastal areas receiving effluents from copper mines. The alga can accumulate high amounts of copper and possesses a strong antioxidant system. Here, we performed short-term transcriptomic analyses using total RNA of the alga cultivated with 10 μM of copper for 0, 3, 6, 12 and 24 h by RNA-seq.

**Results:**

De novo transcriptomes were assembled using the Trinity software, putative proteins were annotated and classified using Blast2GO. Differentially expressed transcripts were identified using edgeR. Transcript levels were compared by paired times 0 vs 3, 0 vs 6, 0 vs 12 and 0 vs 24 h at an FDR < 0.01 and Log2 Fold Change > 2. Up-regulated transcripts encode proteins belonging to photosystem II (PSII), Light Harvesting II Complex (LHCII), PSI and LHCI, proteins involved in assembly and repair of PSII, and assembly and protection of PSI. In addition, transcripts encoding enzymes leading to β-carotene synthesis and enzymes belonging to the Calvin-Benson cycle were also increased. We further analyzed photosynthesis and carotenoid levels in the alga cultivated with 10 μM of copper for 0 to 24 h. Photosynthesis was increased from 3 to 24 h as well as the level of total carotenoids. The increase in transcripts encoding enzymes of the Calvin-Benson cycle suggests that C assimilation may also be increased.

**Conclusions:**

Thus, *U. compressa* displays a short-term response to copper stress enhancing the expression of genes encoding proteins involved in photosynthesis, enzymes involved carotenoids synthesis, as well as those belonging to the Calvin-Benson cycle, which may result in an increase in C assimilation.

**Electronic supplementary material:**

The online version of this article (10.1186/s12864-018-5226-4) contains supplementary material, which is available to authorized users.

## Background

Heavy metals such as zinc, iron, copper, and manganese are essential for plant cell viability since they are associated with proteins and enzymes such as superoxide dismutase, plastocyanin, cytochrome c oxidase, amino oxidase, polyphenol oxidase, nitrogenase, and several dehydrogenases and oxidases [1, 2]. However, excess of essential heavy metals can be toxic to cells since they induce oxidative stress leading to oxidation and destruction of biological macromolecules. On the other hand, some heavy metals and metalloids such as cadmium, lead, mercury, arsenic are not required for enzyme activity and they are always toxic for the cells. In particular, copper is required for photosynthetic activity in the chloroplast since it is an essential component of plastocyanin that allows transfer of electrons from cytochrome b_6_*f* to photosystem I (PSI) reaction center. Copper is also required by cytochrome c oxidase, the final acceptor of electrons of respiratory chain, in the mitochondria and Cu/Zn superoxide dismutase that converts superoxide anions into hydrogen peroxide, mainly in the cytosol [3–5].

Regarding copper excess and photosynthesis in plants and green microalgae, it has been shown that copper toxicity is caused by the replacement of magnesium in chlorophyll by copper ions, which does not allow the efficient release of energy from chlorophyll in antenna complexes to reaction centers in Photosystem II (PSII) or directly inhibits the reaction center of PSII [3–5]. For example, the aquatic plant *Ceratophyl demersum* exposed to nanomolar concentrations of copper for 6 weeks showed an optimal growth at 10–30 nM of copper and an optimal activity of PSII at 2 nM of copper [3]. Conversely, *C. demersum* exposed to 100–200 nM copper showed an inhibition of growth, a decrease in leaf size, fragile stems, chlorosis, and an inhibition of photochemical activity in PSII [3]. Likewise, rice plants cultivated with copper concentrations from 30 nM to 98 μM for 30 days displayed an inhibition of photosynthesis above 157 nM mostly due to inhibition of PSII and decrease in ATP synthase activity [6]. In addition, *Phaseolus vulgaris* plants cultivated with 0.5 to 160 μM of copper for 24 days showed an inhibition of growth and in photosynthesis efficiency [7]. Thus, it appears that plants do not tolerate copper concentrations higher than 100–200 nM due to an inhibition of photosynthesis.

On the other hand, the concentration of copper in seawater in central Chile is around 30 nM whereas in sites of Northern Chile that receive effluents from copper mines, the concentration of copper can reach 300 nM [8]. In these copper-polluted sites, the dominant alga species is the green macroalga *Ulva compressa*, which suggests that this species may tolerate copper excess. In fact, the alga has been cultivated with 10 and 50 μM of copper for 7 days showing no sign of loss in viability; however, at higher concentrations of copper the viability was inhibited [9]. Until now, the effect of increasing copper concentrations on photosynthesis has not been analyzed in *U. compressa*. Regarding other macroalgae species, the brown macroalga *Ectocarpus siliculosus* cultivated with 1.8 μM for 8 h showed an increase in photosynthesis efficiency, whereas it decreased with 3.7 μM of copper [10]. In contrast, the red macroalga *Gracilaria tenuistipitata* cultivated with 16 nM of copper showed a decrease in photosynthesis efficiency after 1, 3 and 6 days of metal exposure [11]. The red macroalga *Porphyra haitiensis* cultivated with 0.1 to 50 μM copper for 3 days showed an increase in photosynthesis when cultivated with 0.1 and 1 μM copper and an increase in respiration when cultivated with 0.1 to 50 μM copper [12]. Furthermore, the green macroalga *Ulva flexuosa* cultivated with 0.8, 4 and 8 μM for 5 days did not show inhibition of photosynthesis [13]. Thus, marine macroalgae appeared to be more tolerant to copper excess than plants since photosynthesis is not inhibited or is increased with micromolar concentrations of copper.

Transcriptomic analyses using RNA-seq and microarrays in marine algae have revealed some specific responses to metals and other abiotic stresses. A microarray analysis of the brown macroalga *E. siliciulosus* cultivated with 1.8 μM copper showed the activation of the oxylipin signaling pathway, the repression of the inositol signaling pathway, an increase or inhibition in expression of some transcription factors and an increase in expression of ABC transporters orthologs, P-type ATPases, ROS-detoxifying enzymes and a vanadium-dependent bromoperoxidase [14]. In addition, *E. siliculosus* treated with copper showed a decrease in nitrogen assimilation, as well as an increase in fatty acid content and autophagy processes. Moreover, some of the up- and down-regulated genes in *E. siliculosus* showed identity to plant genes but a high number corresponded to yet uncharacterized genes [14]. In addition, a transcriptomic analysis performed in *Ulva linza* exposed to low and high temperature, high light, salt and UV-B showed an increase in expression of photoprotective proteins LhcbSR and PsbS and an increase in expression a carbonic anhydrase that improves carbon fixation. In addition, *U. linza* showed an increase in the expression of ammonium, phosphate and sulfate transporters which may improve nutrient uptake and increase in glutamate dehydrogenases, which may improve N fixation. Furthermore, *U. linza* showed an increase in expression of antioxidant enzymes, which may inhibit oxidative stress induced by abiotic stresses [15]. We have previously performed transcriptomic analyses in *U. compressa* cultivated with 10 μM copper for 0 and 24 h and identified seven potential metallothioneins, antioxidant enzymes such as ascorbate peroxidase, glutathione reductase and peroxiredoxin, as well as enzymes involved in glutathione and ascorbate synthesis and their levels appeared to be increased in response to copper stress [16].

In this work, we generated de novo transcriptomes and analyzed short-term differential gene expression in *U. compressa* cultivated with 10 μM of copper for 0, 3, 6, 12 and 24 h. The level of transcripts encoding proteins related with photosynthesis and enzymes of carotenoid synthesis and the Calvin-Benson cycle were increased compared with the control (0 h). To confirm these findings, photosynthesis and the level of carotenoids and chlorophylls were determined in the alga cultivated with 10 μM of copper for 0 to 24 h.

## Results

### *U. compressa* transcriptomes: Assembly, annotation, and classification of transcripts

Sequencing of cDNA libraries prepared from *U. compressa* cultivated without copper (0 h, control) resulted in 11.4 M of reads and those prepared from the alga cultivated with 10 μM copper for 3, 6, 12 and 24 h resulted in 18.4 M of reads on average (Table 1). Reads were trimmed, and bacterial sequences were eliminated using the Bowtie2 software by aligning the reads to bacterial reference genomes from the NCBI-RefSeq database. After quality control, the control condition presented 10.9 M of reads and the copper-treated samples in 11.9 M on average (Table 1; Additional file 1: Figure S1). Transcripts were assembled using the Trinity software and resulted in 237,116 transcripts (contigs) of 101 to 9118 nucleotides in length (Additional file 1: Figure S1). Transcripts having 200 or less nucleotides in length were removed, which resulted in 106,704 total transcripts having an average length of 868 bp and corresponding to 45% of the initial total transcripts (Table 1). The 106,704 transcripts represent 64,191 genes with a N50 value of 1383 bp, average GC content of 58.18%, where 81.59% of transcripts were present in Core Eukaryotic Genes datasets (CEGs and OrthoDB databases) (Table 1).

**Table 1:** Sequencing, pre-processing of reads and assembly of the *Ulva compressa* transcriptome

Libraries	Sequence reads	Reads after QC	Reads after decontamination
Sequencing, quality control and decontamination of reads			
0 h-1	11,438,569	11,152,558 (97.50%)	10,861,476 (94.95%)
3 h-1	20,650,530	15,993,724 (77.45%)	15,768,212 (76.36%)
3 h-2	20,884,112	18,978,764 (90.88%)	18,756,712 (89.81%)
6 h-1	21,666,034	19,744,705 (91.13%)	19,436,688 (89.71%)
6 h-2	19,419,660	17,691,271 (91.10%)	17,383,443 (89.51%)
12 h-1	17,354,946	15,909,292 (91.67%)	15,785,199 (90.96%)
12 h-2	16,308,426	12,485,489 (76.56%)	12,360,634 (72.79%)
24 h-1	12,467,371	12,150,061 (97.45%)	11,862,105 (95.15%)
Transcriptome assembly			
Total genes		64,191	
Total transcripts		106,704 (92.57 Mb)	
Total bases		92,575,573 (92.57 Mb)	
Min sequence lenght		200	
Max sequence lenght		9118	
Average sequence lenght		868	
Median sequence lenght		542	
N25 lenght		2,28	
N50 lenght		1383	
N75 lenght		716	
N90 lenght		349	
N95 lenght		261	
A		20.95%	
T		20.87%	
G		29.18%	
C		29.00%	
(A + T)		41.82%	
(G + C)		58.18%	
N		0.00%	
Transcriptome completeness			
Completeness		89.59%	
Complete CEG		265	
Complete and single copy		123	
Complete and multi-copy		142	
Fragmented CEGs		85	
Missing CEGs		79	
Total CEGs database		429	

**Fig. 1:**
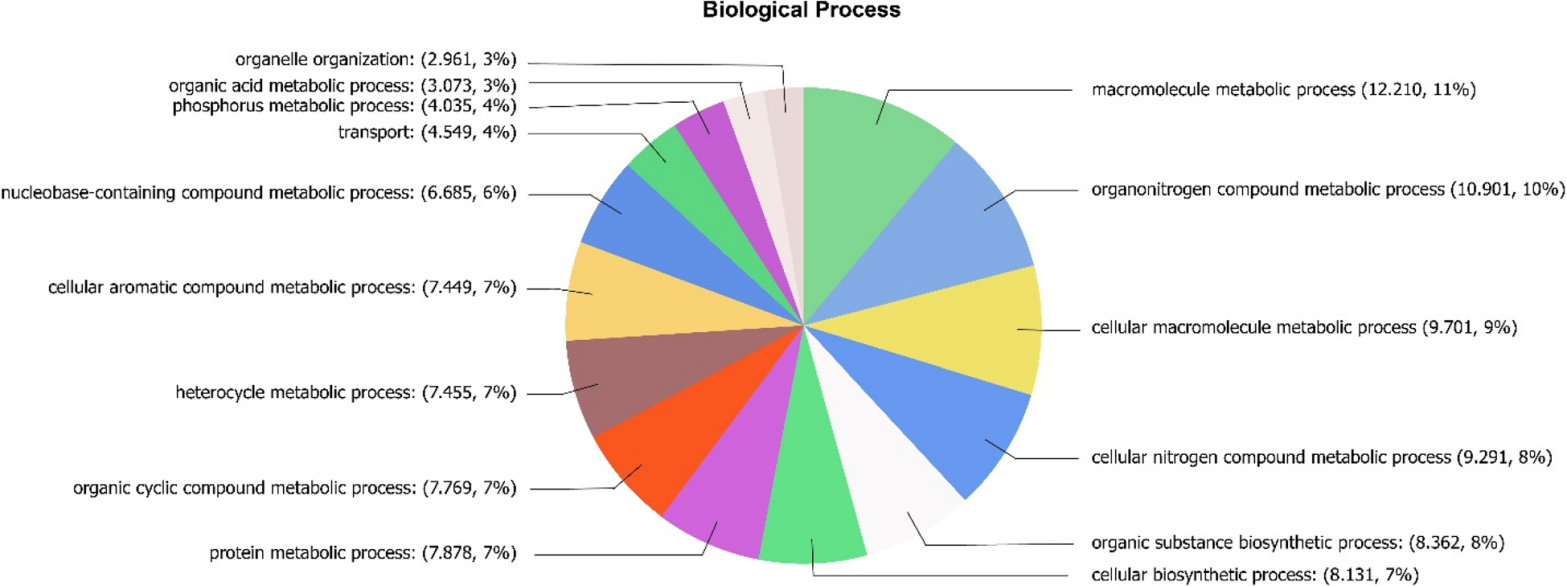
Pie chart of percentage of protein sequences associated with different biological processes obtained from the transcriptomic analyses performed in the marine alga *Ulva compressa* cultivated with 10 μM copper for 0, 3, 6, 12 and 24 h

Transcripts were translated into amino acids using BlastX and those having an e-value of 1e^− 3^ or less were selected. Transcripts were annotated using UniprotKB database with Blat2GO software and 40,037 protein sequences were obtained of which 36,094 were selected having an e-value of 1e^− 6^ or lower. These 36,094 proteins are involved in different biological processes corresponding to: 12.210 (11%) associated with macromolecules metabolic processes; 10.901 (10%) with organic nitrogen metabolic processes; 9.701 (9%) with cellular macromolecule processes; 9.291 (8%) with cellular nitrogen compound metabolic processes; 8.362 (8%) with organic substance biosynthetic processes; 8.131 (7%) with cellular biosynthetic processes; 7.878 (7%) with protein metabolic processes; 7.769 (7%) with organic cyclic metabolic processes; 7.455 (7%) in heterocycle metabolic processes; 7.499 (7%) with aromatic compound metabolic processes; 6.685 (6%) with nucleobase-containing compound metabolic processes; 4.549 (4%) with transport; 4.035 (4%) with phosphorous metabolic processes; 3.073 (3%) with organic acid metabolic processes and 2.961 (3%) with organelle organization processes (Fig. 1). Thus, at least 21% of the proteins are involved in secondary metabolism, 4% in transport and 3% in organelle organization. In addition, proteins were classified according to their molecular function and cellular component (Additional file 2: Figure S2).

**Fig. 2:**
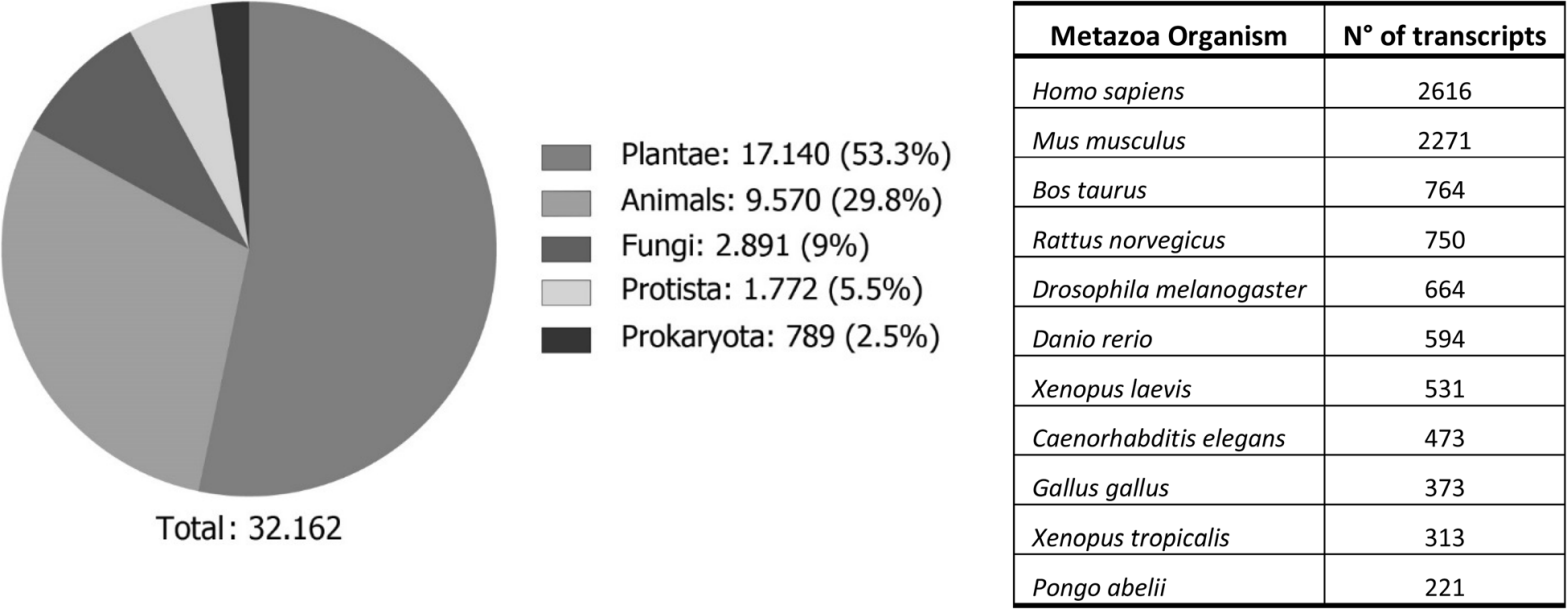
Pie chart of percentage of protein sequences associated with different classes of organisms obtained from the transcriptomic analyses performed in the marine alga *Ulva compressa* cultivated with 10 μM copper for 0, 3, 6, 12 and 24 h

Thirty-two thousand and 162 protein sequences were selected using BLAST Top-Hit species and a database of proteins belonging to 29 model species to analyze the similarity to plant, animal, protist, fungal, and prokaryote proteins. Results indicate that 17,140 proteins (53.3%) are similar to plant proteins; 9570 (29.8%) to animal proteins; 2891 (9%) to fungal proteins; 1772 (5.5%) to protist proteins and 789 (2.5%) to prokaryote proteins (Fig. 2). These results suggest that almost 30% of *U. compressa* proteins are related to animal proteins corresponding to 11 animal species including human, mouse and rat (Fig. 2) and 53% with plant proteins.

### Transcripts differentially expressed in response to copper stress

**Fig. 3:**
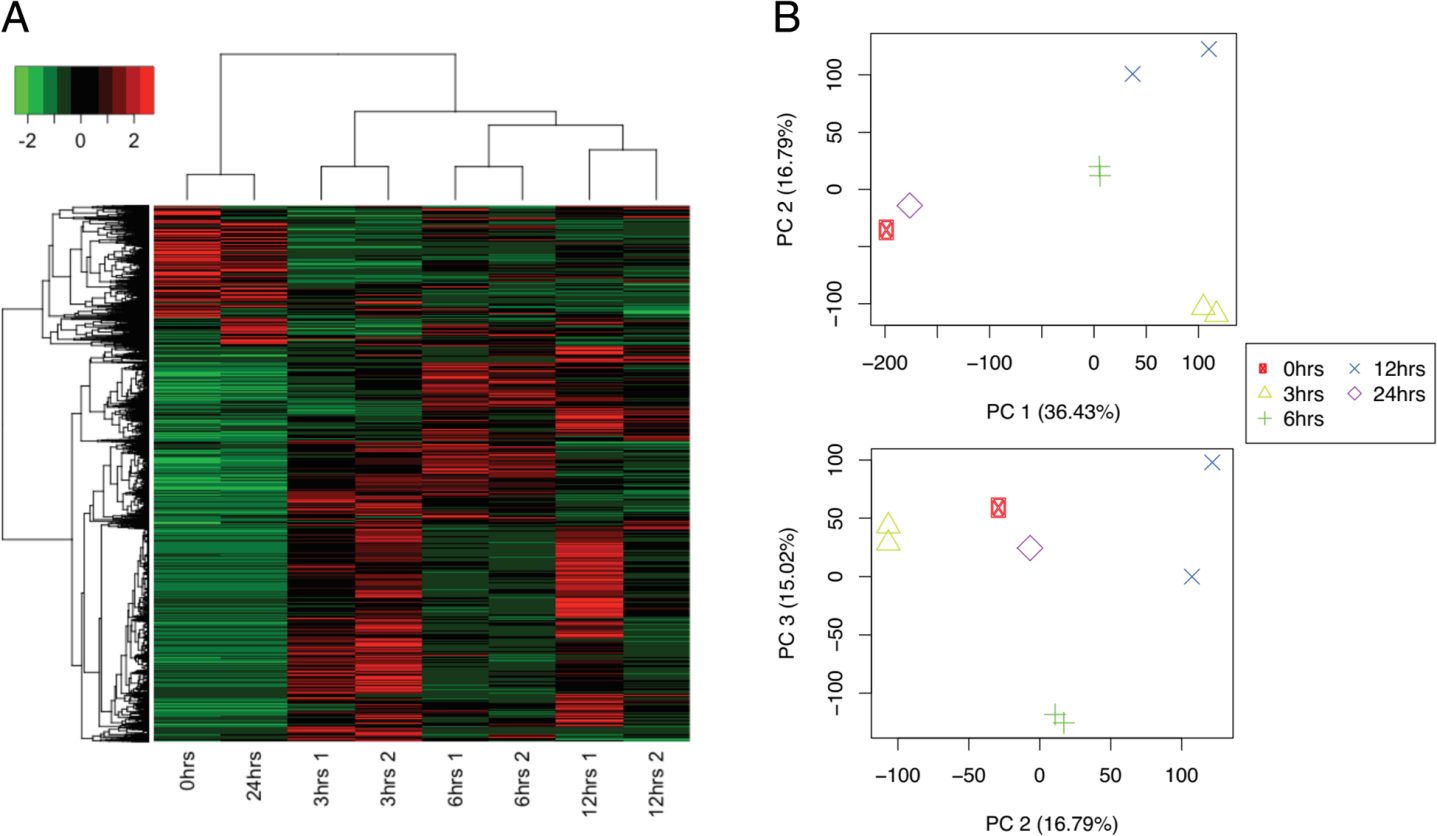
Gene expression profile and sample-to-sample distance relationships. **a** Heatmap representing the expression levels (read counts) of the differentially expressed transcripts (FDR < 0.01, FC > 2) across samples: 0, 3, 6, and 12 h (“3 h 1”, “3 h 2”, “6 h 1”, “6 h2”, “12 h 1” and “12 h 2”). Read counts were mean-centered and scaled, and the resulting values were represented in a green red false color scale (green = underexpressed; red = overexpressed). Columns and rows were clustered by the Spearman correlation coefficient among samples and transcripts, respectively. **b** Principal component analysis (PCA) showing how the samples are grouped based on transcripts CPM (counts per million) values at each sample. Top plot shows the first and second components (53.22% of variance), and bottom plot shows the second and third components (31.81% of variance)

Transcripts were filtered by FDR of 0.01 or lower, and a Log2 Fold Change higher than 2. From 106,704 transcripts, only 23,978 were differentially expressed. When clustering samples by their differential expression profiles, it was observed that samples taken at 0 and 24 h had similar differential expression patterns, while samples at 3, 6, and 12 h showed time-specific profiles. All biological replicates were more similar to each other than to other samples (Fig. 3a). This was also true when comparing the expression profiles of all transcripts, not only those differentially expressed (Fig. 3b). This suggests that copper has a measurable and specific impact upon gene expression, indicating that those differentially expressed genes might reveal molecular mechanisms by which *U. compressa* copes with copper excess.

### Transcripts with increased level encode proteins involved in assembly, repair and protection of photosystems

**Fig. 4:**
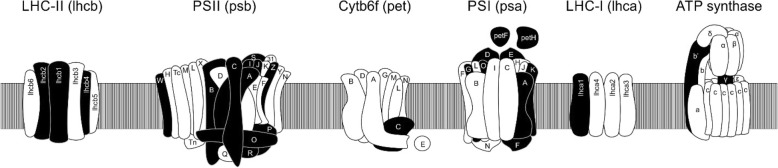
Scheme of Light Harvesting Complex II (LHCII), photosystem II (PSII), cytochrome b6f complex (cytb6f), photosystem I (PSI) and light harvesting complex I (LHCI) in terrestrial plants. Subunits encoding transcripts showing increased levels in the transcriptomic analyses performed in the marine alga *Ulva compressa* cultivated with 10 μM copper for 0, 3, 6, 12 and 24 h are highlighted in black

The level of transcripts encoding subunits PsbA, PsbB, PsbC, PsbD, PsbJ, PsbO, PsbP, PsbR, PsbS, PsbW and PsbZ were increased in response to copper stress (Table 2; Fig. 4). In addition, transcripts encoding proteins belonging to the Light Harvesting Complex II (LHCII) involved in chlorophyll a/b-binding corresponding to Lhcb1, Lhb2 and Lhb4, present in plants, and those encoding proteins involved in fucoxanthin and chlorophyll a/c binding corresponding to LhcbA and LhcB, present in diatoms, were also up-regulated (Table 2; Fig. 4). Moreover, transcripts encoding enzymes solanesyl diphosphate synthase, involved in the synthesis of the terpene tail of plastoquinone as well as iron-sulfur subunit of cytochrome b6f complex (PetC) were increased (Table 2; Fig. 4). Furthermore, transcripts encoding proteins of PSI, PsaA, PsaD, PsaE, PsaF, PsaG, psaK and PsaO, as well as ferredoxin-NADP reductase (PetH) and ferredoxin (PetF) were increased (Table 2; Fig. 4). In addition, transcripts encoding proteins belonging to the Light Harvesting Complex I (LHCI) corresponding to Lhca1, Lhca5 and Lhca8 were also increased in response to copper excess (Table 2; Fig. 4). Finally, transcripts encoding subunit b’ and gamma subunits of ATP synthase complex were also increased (Table 2; Fig. 4).

**Table 2:** Up-regulated genes related to photosynthesis

Process	ID_Transcript	Proteins	Fold change
PSII	TRINITY_DN28356_c0_g7_i2	PsbA	3.3
	TRINITY_DN26114_c0_g9_i1	PsbB	2.0
	TRINITY_DN24036_c0_g1_i1	PsbC	2.5
	TRINITY_DN51574_c0_g1_i1	PsbJ	3.2
	TRINITY_DN25152_c1_g6_i1	PsbO	3.0
	TRINITY_DN25152_c1_g3_i1	PsbO	2.8
	TRINITY_DN23656_c0_g2_i2	PsbP	2.7
	TRINITY_DN30969_c0_g1_i1	PsbP	2.9
	TRINITY_DN134452_c0_g1_i1	PsbR	2.9
	TRINITY_DN24151_c1_g3_i1	PsbS	2.2
	TRINITY_DN22768_c0_g2_i1	PsbW	3.0
	TRINITY_DN21681_c1_g1_i2	PsbW	3.3
	TRINITY_DN21681_c1_g1_i1	PsbW	3.2
	TRINITY_DN22451_c0_g1_i5	PsbZ	2.8
LHCII	TRINITY_DN28384_c2_g10_i1	Chlorophyll a-b binding protein 1D (lhcb1)	3.3
	TRINITY_DN24974_c1_g9_i1	Chlorophyll a-b binding protein 1D (lhcb1)	2.9
	TRINITY_DN28161_c1_g6_i2	Chlorophyll a-b binding protein 8 (lhcb2-like)	3.2
	TRINITY_DN23399_c0_g1_i2	Chlorophyll a-b binding protein CP29 (lhcb4)	2.9
	TRINITY_DN23399_c0_g1_i3	Chlorophyll a-b binding protein CP29 (lhcb4)	2.9
	TRINITY_DN23399_c0_g1_i1	Chlorophyll a-b binding protein CP29 (lhcb4)	2.8
	TRINITY_DN25769_c2_g3_i4	Chlorophyll a-b binding protein L1818 (lhcb4-like)	3.4
	TRINITY_DN25769_c2_g3_i2	Chlorophyll a-b binding protein L1818 (lhcb4-like)	3.1
	TRINITY_DN23036_c1_g1_i8	Fucoxanthin binding protein A	2.8
	TRINITY_DN23036_c1_g1_i5	Fucoxanthin binding protein A	2.8
	TRINITY_DN28382_c2_g1_i8	Fucoxanthin binding protein B	3.0
	TRINITY_DN28382_c2_g1_i7	Fucoxanthin binding protein B	2.7
	TRINITY_DN25548_c0_g1_i1	Solanesyl diphosphate synthase 1	3.2
	TRINITY_DN25548_c0_g1_i2	Solanesyl-diphosphate synthase 3	3.3
	TRINITY_DN25548_c0_g1_i3	Solanesyl-diphosphate synthase 3	3.2
	TRINITY_DN25548_c0_g1_i7	Solanesyl-diphosphate synthase 3	3.4
Cyt b6-f	TRINITY_DN27161_c0_g1_i10	Iron-sulfur subunit (petC)	3.7
	TRINITY_DN27161_c0_g1_i1	Iron-sulfur subunit (petC)	3.1
	TRINITY_DN27161_c0_g1_i7	Iron-sulfur subunit (petC)	2.7
	TRINITY_DN27161_c0_g1_i13	Iron-sulfur subunit (petC)	2.6
	TRINITY_DN27161_c0_g1_i12	Iron-sulfur subunit (petC)	2.5
PSI	TRINITY_DN26298_c1_g1_i5	PsaA	3.4
	TRINITY_DN26298_c1_g1_i1	PsaA	3.2
	TRINITY_DN26298_c1_g1_i6	PsaA	3.0
	TRINITY_DN26298_c1_g1_i3	PsaA	2.6
	TRINITY_DN24286_c0_g3_i1	PsaD	1.9
	TRINITY_DN21945_c0_g2_i1	PsaE	3.3
	TRINITY_DN23092_c0_g3_i1	PsaF	3.6
	TRINITY_DN23521_c0_g1_i1	PsaG	3.6
	TRINITY_DN23521_c0_g1_i2	PsaG	3.3
	TRINITY_DN25932_c0_g1_i3	PsaK	3.6
	TRINITY_DN21897_c0_g2_i1	PsaO	3.4
	TRINITY_DN26200_c0_g3_i1	Ferredoxin-NADP reductase (petH)	2.9
	TRINITY_DN24658_c0_g1_i1	Ferredoxin-2 (petF)	2.8
LHCI	TRINITY_DN24974_c1_g7_i2	Chlorophyll a-b binding protein 1	2.8
	TRINITY_DN28384_c2_g5_i2	Chlorophyll a-b binding protein 1	3.3
	TRINITY_DN28268_c0_g2_i6	Chlorophyll a-b binding protein 1	3.2
	TRINITY_DN28268_c0_g2_i9	Chlorophyll a-b binding protein 1	3.0
	TRINITY_DN28268_c0_g2_i7	Chlorophyll a-b binding protein 1	2.9
	TRINITY_DN28384_c2_g17_i1	Chlorophyll a-b binding protein 1	2.8
	TRINITY_DN26721_c1_g6_i1	Chlorophyll a-b binding protein 1B-21 (lhca1)	3.2
	TRINITY_DN26721_c1_g6_i2	Chlorophyll a-b binding protein 1B-21 (lhca1)	3.5
	TRINITY_DN27296_c2_g4_i2	Chlorophyll a-b binding protein 5 (lhca1-like)	2.6
	TRINITY_DN28268_c0_g2_i3	Chlorophyll a-b binding protein 5 (lhca1-like)	2.5
	TRINITY_DN25309_c0_g1_i2	Chlorophyll a-b binding protein 5 (lhca1-like)	2.5
	TRINITY_DN27296_c2_g1_i11	Chlorophyll a-b binding protein 5 (lhca1-like)	2.2
	TRINITY_DN25604_c0_g1_i1	Chlorophyll a-b binding protein 5 (lhca1-like)	2.6
	TRINITY_DN25604_c0_g1_i4	Chlorophyll a-b binding protein 5 (lhca1-like)	3.4
	TRINITY_DN28161_c1_g6_i3	Chlorophyll a-b binding protein 8 (lhca1-like)	2.1
	TRINITY_DN28161_c1_g6_i4	Chlorophyll a-b binding protein 8 (lhca1-like)	2.0
ATP synthase	TRINITY_DN23229_c0_g3_i1	ATP synthase subunit b’	3.4
	TRINITY_DN24803_c0_g1_i1	ATP synthase gamma chain	1.4
Repair of PSII	TRINITY_DN26385_c1_g4_i3	MET1	3.5
	TRINITY_DN25782_c0_g1_i2	MET1	3.4
	TRINITY_DN26385_c1_g4_i2	MET1	3.3
	TRINITY_DN27340_c1_g3_i2	Deg/HtrA protease Do-like 1	3.3
	TRINITY_DN26676_c1_g1_i1	Deg/HtrA protease Do-like 1	3.3
	TRINITY_DN27340_c1_g3_i3	Deg/HtrA protease Do-like 1	3.2
	TRINITY_DN26676_c1_g1_i12	Deg/HtrA protease Do-like 1	3.2
	TRINITY_DN27340_c1_g3_i4	Deg/HtrA protease Do-like 1	3.2
	TRINITY_DN27340_c1_g3_i1	Deg/HtrA protease Do-like 1	3.1
	TRINITY_DN25574_c0_g1_i4	Deg/HtrA protease Do-like 2	2.9
	TRINITY_DN25574_c0_g1_i7	Deg/HtrA protease Do-like 2	2.7
	TRINITY_DN18665_c0_g1_i1	Deg/HtrA protease Do-like 5	3.0
	TRINITY_DN28204_c1_g1_i2	Deg/HtrA protease Do-like 8	2.7
	TRINITY_DN25743_c0_g2_i1	EGY1	3.2
	TRINITY_DN25743_c0_g2_i2	EGY1	3.0
	TRINITY_DN25743_c0_g2_i3	EGY1	2.9
	TRINITY_DN23341_c0_g1_i1	ATP-dependent zinc metalloprotease FTSH 1	3.2
	TRINITY_DN27493_c2_g1_i7	ATP-dependent zinc metalloprotease FTSH 2	3.1
	TRINITY_DN27493_c2_g1_i1	ATP-dependent zinc metalloprotease FTSH 2	3.1
	TRINITY_DN27493_c2_g1_i11	ATP-dependent zinc metalloprotease FTSH 2	3.1
	TRINITY_DN28584_c2_g1_i4	ATP-dependent zinc metalloprotease FTSH 5	3.3
	TRINITY_DN27493_c2_g4_i1	ATP-dependent zinc metalloprotease FTSH 8	2.9
	TRINITY_DN27493_c2_g4_i3	ATP-dependent zinc metalloprotease FTSH 8	2.9
	TRINITY_DN24421_c0_g1_i1	THYLAKOID FORMATION 1	3.2
	TRINITY_DN22196_c0_g2_i1	EMBRYO SAC DEVELOPMENT ARREST 3	2.9
	TRINITY_DN25705_c1_g2_i2	Palmitoyl-monogalactosyldiacylglycerol delta-7 desaturase	2.6
Assembly of PSII	TRINITY_DN23616_c0_g1_i4	YCF4	3.3
	TRINITY_DN23616_c0_g1_i2	YCF4	2.1
	TRINITY_DN22135_c0_g2_i3	Ycf3 interacting Protein	2.9
	TRINITY_DN27520_c0_g2_i3	KEA1	2.7
	TRINITY_DN24215_c0_g1_i3	UPF0187 protein	2.7
	TRINITY_DN25274_c0_g1_i1	TAB2 homolog	3.2
	TRINITY_DN25675_c1_g3_i1	Serine/threonine-protein kinase STN7	2.7
	TRINITY_DN25616_c0_g2_i1	PPH1 2C 57	3.1
	TRINITY_DN28540_c1_g8_i2	PGR5 1A	3.5
	TRINITY_DN28540_c1_g2_i2	PGR5 1A	3.1
	TRINITY_DN27605_c0_g1_i2	PGR5 1A	2.9
	TRINITY_DN26853_c0_g1_i1	PGR5 1B	3.3
	TRINITY_DN20751_c0_g2_i1	PGR5	3.3

Transcripts encoding proteins involved in repair of PSII were also up-regulated such as MET1, involved disassemble and reassemble of PSII to remove damaged PsbA (D1). In addition, the serine proteases Deg/HtrA (Do)-like proteases 1, 2, 5 and 8, the metalloprotease EGY1 and the ATP-dependent proteases FTSH 1, 2, 5 and 8 involved in the degradation of damaged PsbA, and other oxidized subunits of PSII, were also up-regulated (Table 2). Moreover, transcripts encoding proteins involved in chloroplast repair such as Thylakoid Formation 1 (THF1), involved in the formation of the supramolecular complex among PSII and LHCII, Embryo Sac Development Arrests 3 (ESD3), involved in thylakoid formation, and the enzyme palmitoyl-monogalactosyl diacylglycerol delta 7-desaturase involved in the synthesis of chloroplast fatty acids were also increased (Table 2). On the other hand, transcripts encoding proteins involved in assembly and stabilization of PSI such as Ycf-4 and Ycf-3-interacting protein that cooperate with Ycf3 in the assembly of PSI were enhanced. In addition, the potassium antiporter KEA1, required for PSI formation, UPF0187, involved in the stabilization of PSI, TAB2, involved in translation of PsaB, the kinase STN7, involved in phosphorylation of the LHCII that allows its migration of subunits of PSII to PSI, and the phosphatase PPH1 (2C57), involved in the dephosphorylation of LHCII that increase the migration of Lhcb1 and Lhcb2 subunits to PSI, were also increased. Furthermore, PGR1 and PGR5, two proteins involved in the control of the cyclic electron flow around PSI and protecting PSI against photo-oxidation were also up-regulated (Table 2).

### Transcripts with increased levels encode enzymes involved in carotenoid synthesis and the Calvin-Benson cycle

The level of transcripts encoding the enzymes geranylgeranyl diphosphate synthase (GGDS), an enzyme that synthesize geranylgeranyl pyrophosphate; phytoene synthase (PS), that synthesize phytoene; phytoene desaturase (PD), that synthesize ϛ-carotene; carotene desaturase (CD), that synthesize lycopene, and lycopene β-cyclase (LC), that synthesize β-carotene were increased (Table 3). In addition, the level of transcripts encoding enzymes of the Calvin-Benson cycle such as the small subunit (RbcS) of ribulose 1,5 biphosphate carboxylase/oxygenase (rubisco), ribulose phosphate 3 epimerase (RP3E), transketolase (TK), glyceraldehyde 3 phosphate dehydrogenase (G3PDH), fructose 1,6 biphosphatase (FBP), sedoheptulose 1,7 biphosphatase (SHBP), phosphoribulokinase (PRK), phosphoglycerate kinase (PGK) and ribose 5-phophate isomerase (R5PI) were also up-regulated (Table 3).

**Table 3:** Up-regulated genes related to Calvin-Benson cycle

Process	ID_Transcript	Proteins	Fold change
Calvin-Benson cycle	TRINITY_DN26739_c1_g1_i11	Ribulose bisphosphate carboxylase small chain 1	3.5
	TRINITY_DN26739_c1_g1_i6	Ribulose bisphosphate carboxylase small chain 1	3.4
	TRINITY_DN26739_c1_g1_i10	Ribulose bisphosphate carboxylase small chain 1	3.2
	TRINITY_DN26739_c1_g1_i1	Ribulose bisphosphate carboxylase small chain 1	3.0
	TRINITY_DN26739_c1_g1_i8	Ribulose bisphosphate carboxylase small chain 1	3.1
	TRINITY_DN24974_c1_g8_i2	Ribulose-phosphate 3-epimerase	3.2
	TRINITY_DN24974_c1_g8_i1	Ribulose-phosphate 3-epimerase	3.1
	TRINITY_DN24974_c1_g5_i2	Ribulose-phosphate 3-epimerase	3.0
	TRINITY_DN24974_c1_g5_i1	Ribulose-phosphate 3-epimerase	2.9
	TRINITY_DN26523_c0_g6_i1	Transketolase-2	3.4
	TRINITY_DN26523_c0_g5_i1	Transketolase	2.3
	TRINITY_DN26020_c1_g2_i2	Glyceraldehyde-3-phosphate dehydrogenase	3.2
	TRINITY_DN28111_c0_g1_i3	Glyceraldehyde-3-phosphate dehydrogenase	3.2
	TRINITY_DN28111_c0_g6_i2	Glyceraldehyde-3-phosphate dehydrogenase	3.1
	TRINITY_DN25377_c1_g4_i1	Glyceraldehyde-3-phosphate dehydrogenase	3.0
	TRINITY_DN28111_c0_g1_i5	Glyceraldehyde-3-phosphate dehydrogenase	2.7
	TRINITY_DN25377_c0_g1_i1	Glyceraldehyde-3-phosphate dehydrogenase	2.7
	TRINITY_DN25438_c0_g3_i2	Fructose-1,6-bisphosphatase	3.2
	TRINITY_DN25438_c0_g2_i2	Fructose-1,6-bisphosphatase	3.2
	TRINITY_DN25438_c0_g2_i1	Fructose-1,6-bisphosphatase	3.1
	TRINITY_DN25498_c1_g1_i1	Seudoheptulose-1,7-bisphosphatase	3.2
	TRINITY_DN25498_c1_g2_i2	Seudoheptulose-1,7-bisphosphatase	3.0
	TRINITY_DN28545_c0_g3_i2	Phosphoribulokinase	3.0
	TRINITY_DN24724_c0_g2_i4	Phosphoribulokinase	2.7
	TRINITY_DN20744_c0_g1_i2	Phosphoglycerate kinase	2.9
	TRINITY_DN19258_c0_g2_i1	Pyruvate phosphate dikinase	2.9
	TRINITY_DN134423_c0_g1_i1	Pyruvate phosphate dikinase	2.9
	TRINITY_DN28070_c1_g1_i2	Ribose-5-phosphate isomerase 3	3.1
	TRINITY_DN25979_c1_g4_i1	BC1 complex kinase 1	3.1
	TRINITY_DN25979_c1_g4_i2	BC1 complex kinase 1	3.1
	TRINITY_DN24548_c0_g4_i1	Geranylgeranyl diphosphate synthase	2.9
	TRINITY_DN25865_c0_g2_i3	Phytoene synthase	3.1
	TRINITY_DN23375_c0_g1_i3	15-cis-phytoene desaturase	3.0
	TRINITY_DN25489_c0_g1_i1	Zeta-carotene desaturase	3.3
	TRINITY_DN22033_c0_g1_i1	Lycopene beta cyclase	3.0

### Kinetics of the increase of transcripts encoding proteins of photosystems, and enzymes of carotenoid synthesis and Calvin-Benson cycle

**Fig. 5:**
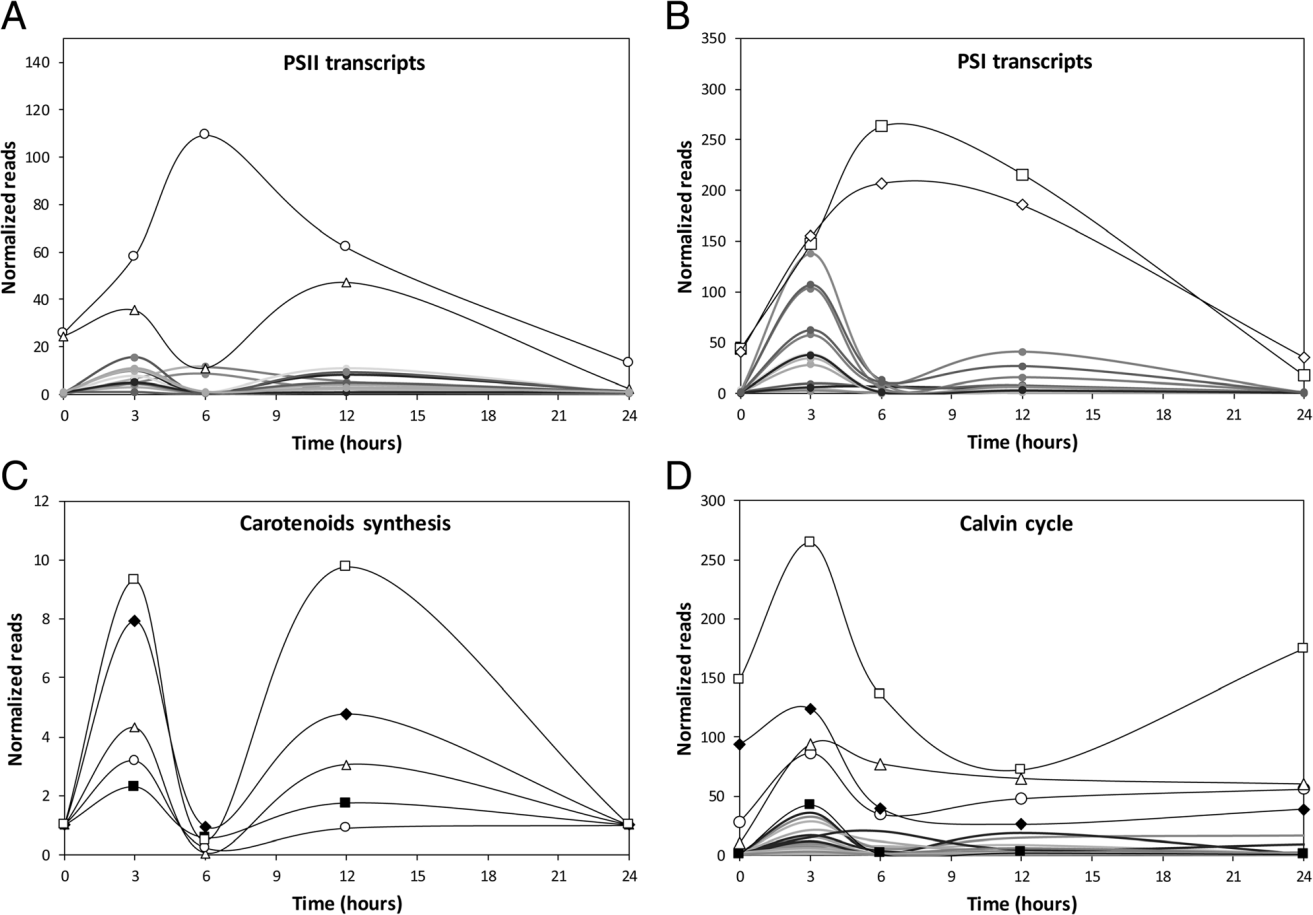
Level of up-regulated transcripts encoding: PsbA (open circles) and PsbW (open triangles) of PSII (**a**), PGR-1A (open squares) of PSI (**b**) and enzymes GGDPS (open squares), CD (black diamonds), PYS (open triangles), LC (open circles) and PD involved in β-carotene synthesis (**c**) and enzymes G3PDH (open squares), RP3E (black diamonds, PRK (open triangles) and TK (open circles) of Calvin-Benson cycle (**d**) in the marine alga *Ulva compressa* cultivated with 10 μM copper for 0, 3, 6, 12 and 24 h. Level of transcripts is expressed as normalized reads and time in hours

The levels of transcripts encoding the subunits of PSII showing the higher increases were those encoding the core subunit PsbA (D1) that increased with a maximal level at 6 h of copper exposure and slowly decreased until 24 h of exposure and the subunit PsbW, a small subunit of 6.1 kDa closely associated with PSII reaction center, that showed maximal increases at 3 and 12 h of copper exposure (Fig. 5a). In the case of transcripts related to PSI, the higher increases were those encoding PGR5-1A that stimulate electron transfer from ferredoxin to plastoquinone and control the electron flow around PSI, and their level increased at 6 h and decreased until 24 h (Fig. 5b). Regarding carotenoids synthesis, transcripts encoding GGDS, PS, PD, CD and LC showed an increase at 3 h of copper exposure and a subsequent increase at 12 of exposure (Fig. 5c). Regarding enzymes of the Calvin-Benson cycle, transcripts encoding G3PDH increased at 3 h of copper exposure, decreased and then increased again at 24 h, and those encoding RP3E, PRK, TK and the small chain of rubisco increased at 3 h of exposure and decrease and remained until 6 h and remained stable until 24 h (Fig. 5d).

### Copper-induced increase in photosynthesis and carotenoid levels

**Fig. 6:**
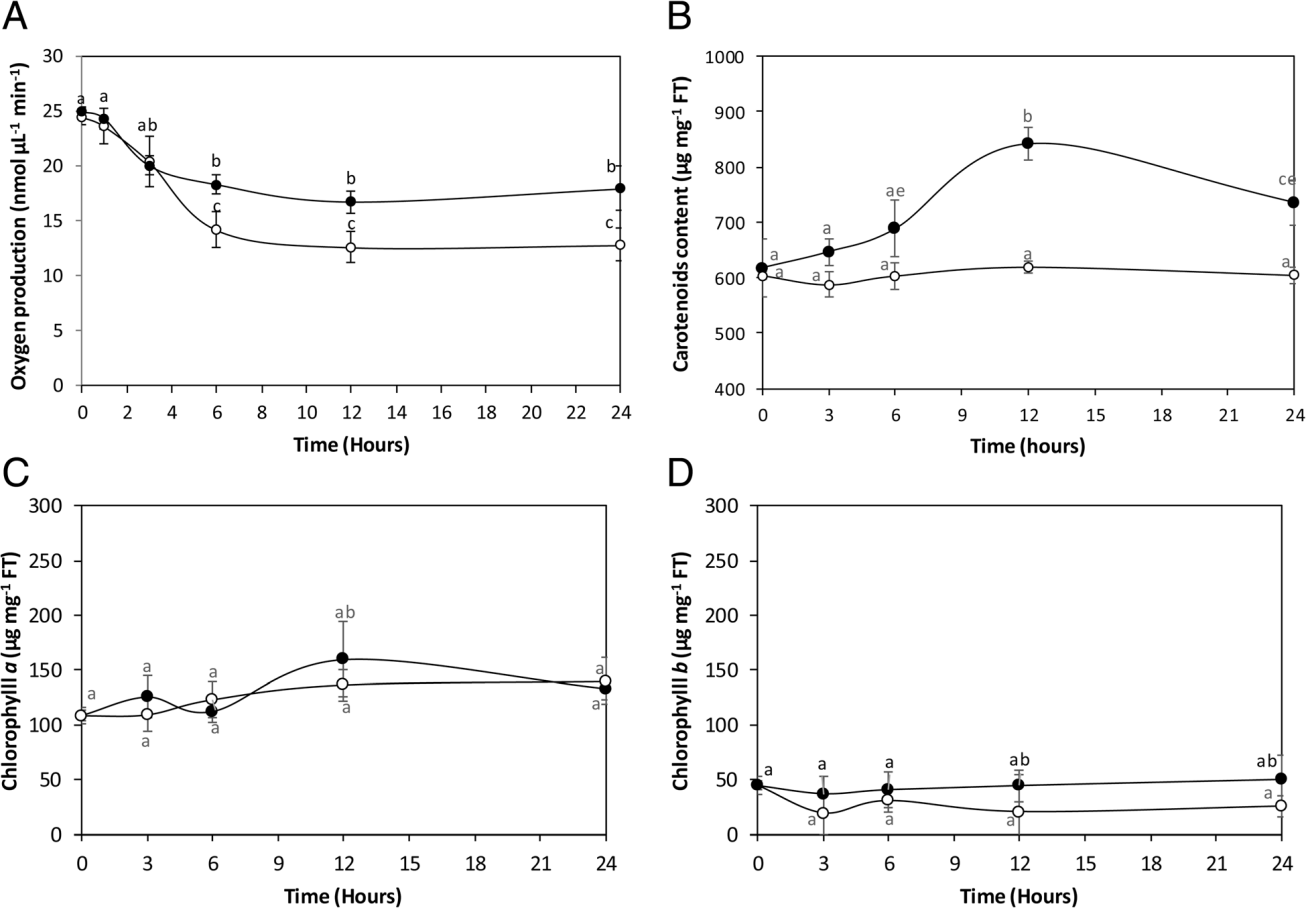
Level of oxygen production (**a**), carotenoids (**b**), chlorophyll *a* (**c**) and chlorophyll *b* (**d**) in the marine alga *Ulva compressa* cultivated in seawater without copper addition (open circles) and with 10 μM copper (black circles) for 0 to 24 h. The level of oxygen is expressed as nanomoles per microliter per minute, the level of carotenoids is expressed in micrograms per milligram of fresh tissue (FT) and time is expressed in hours. Symbols represent mean values of three independent experiments ± SD. Different letters indicate significant differences (*P* < 0.05)

The increase in transcripts encoding subunits of PSII and PSI as well as repair proteins of PSII and assembly and protection of PSI suggests that photosynthesis may be increased in *U. compressa* exposed to copper excess until 24 h. In fact, the alga cultivated in control conditions displayed an oxygen production of 25 nmoles μL^− 1^ min^− 1^ that decreased reaching a level of 14.2 nmoles μL^− 1^ min^− 1^ at 6 h of exposure and continued to decrease until 24 h reaching a level of 12.8 nmoles μL^− 1^ min^− 1^ at 24 h (Fig. 6a). In contrast, the alga exposed to 10 μM copper showed an initial decrease in oxygen production, but it level remained higher than in control conditions reaching a level of 18.3 nmoles μL^− 1^ min^− 1^ at 6 h of exposure and 17.9 nmoles μL^− 1^ min^− 1^ at 24 h, which represents an increase of 1.4 times compared to the control at 24 h (Fig. 6a).

The level of carotenoids in the alga cultivated in control conditions was 618 μg mg^− 1^ of FT, which remained unchanged until 24 h (Fig. 6b). In the alga treated with copper, the level of carotenoids increased reaching a level of 841 μg mg^− 1^ of FT at 6 h of exposure and decreased until at 24 of exposure reaching a level of 735 μg mg^− 1^ of FT which represents an increase 1.2 times compared to the control at 24 h (Fig. 6b). In addition, the level of chlorophylls *a* and *b* (Fig. 6c-d) did not change in the alga exposed to copper excess compare to the control condition. The level of chlorophyll a was 108 μg mg^− 1^ in control and treated samples at time 0 and it was 139 μg mg^− 1^ at 24 h of exposure (Fig. 6c). The level of chlorophyll b was 45 μg mg^− 1^ at time 0 in control and treated samples; this level was 26 μg mg^− 1^ in control samples and 50 μg mg^− 1^ in treated samples at 24 h of exposure but these levels were not significantly different (Fig. 6d). Thus, copper stress induced an increase in photosynthesis and in the level of carotenoids in *U. compressa* that is regulated, at least in part, at transcriptional level.

## Discussion

In this work, we showed that the marine alga *U. compressa* cultivated with 10 μM copper for 24 h displays an increase in transcripts encoding subunits of PSII, LHCII, PSI and LHCI, as well as in proteins and enzymes involved in the assembly and repair of PSII, and in the assembly and protection of PSI. The increase in transcripts encoding proteins involved in assembly, protection and repair photosystems, correlated with an increase in photosynthesis in the alga cultivated with 10 μM copper for 0 to 24 h. Thus, copper induces an increase in photosynthesis which is due, at least in part, to an increase in gene expression. The latter results are in agreement with those obtained in the red alga *Porphyra haitiensis* that showed an increase in photosynthesis when cultivated with 0.1 and 1 μM copper for 3 days [12], the brown macroalga *E. siliculosus* cultivated with 1.8 μM for 8 h that displayed an increase in PSII efficiency [10] and the green macroalga *Ulva flexuosa* cultivated 8 μM for 5 days that did not showed inhibition of photosynthesis efficiency [13]. Thus, it appeared that red, brown and green marine macroalgae are able to tolerate micromolar concentrations of copper by protecting photosynthesis, but the level of tolerance to copper excess depends on the algal species.

In addition, the increase in photosynthesis observed in *U. compressa* agrees with the increase in the level of carotenoids located in LHCII and LHCI. It is important to mention that increased transcripts encode all the enzymes required for the synthesis β-carotene from isopentenyl diphosphate. It is important to mention that carotenoids are involved in the protection of photosynthetic apparatus by quenching directly chlorophyll triplet states, thus, inhibiting singlet oxygen formation. In contrast to the increase in carotenoids, no changes in the level of chlorophylls *a* and *b* were observed in *U. compressa* exposed to copper stress. On the other hand, the increase in photosynthesis may increase NADPH levels which may enhance the activities of enzymes belonging to the Calvin cycle leading to an increase in C assimilation. In addition, it was observed that at least 21% of transcripts are involved in secondary metabolism suggesting that the increase in C assimilation may be channeled to the synthesis of secondary metabolites such as phenolic compounds and terpenes. In addition, it is possible that N assimilation may also be increased in the alga cultivated with copper since N is required to repair damaged proteins or compounds that contain N, but this assumption will be analyzed in the future.

Another interesting aspect deduced from the transcriptomic analyses is that almost 30% of the transcripts encode proteins having similarity to animal proteins corresponding to 11 model species that includes human, mouse and rat. In this sense, it has been shown in previous works that lipoxygenase in *U. compressa* uses arachidonic acid as substrate, as in animals, instead of linolenic or linoleic acids, as in plants [9]. In addition, *U. compressa* possess functional Transient Receptor Potential (TRP) channels that are normally present in vertebrate and invertebrates, but not in plants [16–18]. Moreover, *U. compressa* exhibits functional Voltage-Dependent Calcium Channels (VDCCs) that allow extracellular calcium entry leading to intracellular calcium release, as animal cells [19]. Furthermore, the release of calcium from endoplasmic reticulum (ER) occurs via NAADP-, IP_3_- and ryanodine-dependent channels *in U. compressa* exposed to copper excess [20] and NAADP- and ryanodine-dependent channels are not present in plants. In this sense, we have previously postulated that the marine macroalga *U. compressa* may contain an ancestral pool of genes and that part of these genes were lost in the animal lineage and another part was lost in photosynthetic organisms during evolution [16–18].

It is important to mention that only few transcriptomic or microarray analyses have been performed in marine macroalgae in response to copper stress and none of them reported a clear increase in the expression of genes encoding proteins involved in photosynthesis. In this sense, the brown macroalga *E. siliciulosus* cultivated with 1.8 μM copper showed an increase in photosynthesis efficiency but only the level of a transcript encoding L1818 (Lhc4-like protein) appeared to be enhanced [10]. In contrast, *E. siliculosus* showed an increase in transcripts of genes encoding enzymes of the oxylipin signaling pathway, a decrease in transcripts of the inositol signaling pathway and a modulation in the level of transcripts encoding some transcription factors in response to copper stress [10]. In addition, *E. siliculosus* exposed to copper displayed an increase in the level of transcripts encoding ABC transporters orthologs, P-type ATPases, ROS-detoxifying enzymes and a vanadium-dependent bromoperoxidase [10]. On the other hand, the green macroalga *U. linza* exposed to low and high temperature, high light, salt and UV-B, but not to copper stress, showed an increase in expression of photoprotective proteins LhcbSR and PsbS and an increase in expression a carbonic anhydrase, that improves carbon fixation. Moreover. *U. linza* exposed to abiotic stresses displayed an increase in the level of transcripts encoding glutamate dehydrogenases, that improves N fixation, and in the level of transcripts coding for antioxidant enzymes, that inhibits oxidative stress [15]. Thus, this is the first case of a marine macroalga showing a clear increase expression of LCH and PS subunits as well as in expression of enzymes involved in C assimilation and carotenoid synthesis in response to copper stress.

## Conclusions

Here, we showed that the marine alga *U. compressa* exposed to copper stress exhibits a short-term gene expression response by enhancing the transcription of genes encoding proteins involved in assembly, repair and protection of PSII and PSI, and enzymes involved in carotenoid synthesis which is reflected in an increase in photosynthesis and carotenoid level. In addition, transcripts encoding enzymes from the Calvin-Benson cycle were also increased which may reflect an enhanced C assimilation.

## Methods

### Alga and seawater sampling

*U. compressa* was collected in Cachagua, a site with no history of metal pollution in central Chile (32° 34’S), transported to the laboratory in plastic bags at 4 °C inside a cooler. Algae were rinsed three times with filtered seawater obtained in Quintay (33° 12’S), a pristine site, cleaned manually and sonicated for 3 min in an ultrasound bath (Branson 3200, Danbury, CT, USA) in order to aid removing epiphytic bacteria and organic debris.

### In vitro cultures

*U. compressa* (100 mg of fresh tissue) was cultivated in seawater with no nutrients addition without copper (control, 0 h) or with 10 μM of CuCl_2_ for 3, 6, 12 and 24 h under an irradiance of 50 μmoles m-^2^ s^− 1^ on a photoperiod of 14 h light: 10 h dark, at 14 °C. All samples were performed in duplicate except 0 and 24 h that were unique sample. All samples were washed with 2 mL of 100 mM Tris-10 mM EDTA pH 7.0, twice for 10 min, in order to eliminate copper ions bound to the cell wall. Samples were dried with paper, frozen in liquid nitrogen and stored at − 80 °C.

### RNA extraction, preparation of cDNA libraries, and sequencing

Total RNA at 0, 3, 6, 12 and 24 h samples was isolated using EZNA total RNA Kit I (Omega Biotek, GA, USA). *U. compressa* (100 mg of each sample) was frozen in liquid nitrogen and homogenized in 1 mL of TRK buffer with 20 uL 2-mercaptoethanol. The samples were centrifuged, the supernatant recovered, mixed with ethanol 70% and transfered to HiBind RNA mini column and washed with RNA Wash Buffer I and II. Finally, total RNA was eluted with 50 μL DEPC water. RNA samples were cleaned with GeneJet RNA Cleanup and Concentration Micro Kit (Thermo, MS, USA). Total RNA integrity was evaluated using a Fragment Analyzer and the software PROsize (Advanced Analytical, Iowa, USA). RNA samples were sended to BGI genomic center (Shenzhen, China), paired end cDNA libraries were prepared and sequenced using a Hi-Seq Illumina 4000.

### De novo assembly and annotation

Reads obtained by RNA-seq were trimmed using Prinseq (version 0.20.4; −min_len 50 -min_qual_mean 20 -ns_max_n 1 -derep 14 -derep_min 9 -lc_method dust -lc_threshold 49 -trim_left 10 -trim_qual_right) and the quality controlled reads were visualized in Fastqc [21]. De novo transcriptomes were assembled using all the samples in Trinity (−-min_contig_length 200) [22]. Sequences were blasted using BlastX software and UniprotKB/Swiss-Prot database, filtering hits with an e-value cutoff of 1 e^− 3^. Annotated sequences were classified according to their Gene Ontology using Blast2GO software [23] and those having an e-value of 1e^− 6^ were selected. Sequences were classified according to GO domain (biological process, molecular function and cellular component) using Blast2GO online search.

### Detection of differentially expressed transcripts

Quality controlled reads were mapped against the de novo transcriptome using Bowtie2 (version 2.2.9; default settings). Raw reads were counted using eXpress (version 1.5.1; default settings) and were then normalized to CPM units using Trinity’s script abundance_estimates_to_matrix.pl under default settings [24]. Differentially expressed transcripts were identified as implemented in EdgeR (3.20.2) at an FDR < 0.01 and Log2 Fold Change > 2, with Trinity’s script run_DE_analysis_from_samples_file.pl [25]. Differentially expressed transcripts were obtained by contrasting sample time points: 0 vs. 3; 0 vs. 6; 0 vs.12; 0 vs. 24 h. Differentially expressed transcripts were visualized as a heatmap by estimating Spearman’s correlation coefficient on transcripts and samples, and hierarchical clustering (average).

### Kinetics of transcripts encoding proteins of photosystems, and enzymes of carotenoid synthesis and Calvin-Benson cycle

Transcripts encoding proteins of photosystems, and enzymes of carotenoid synthesis and Calvin-Benson cycle were selected based on the annotation and GO domain (biological process). Reads of selected transcripts were normalized using Trimmed Mean of M-value (TMM) normalization method [26] they were analyzed by Multiple Experiment Viewer (MeV 4.8.1 version) software [27], groups of transcripts showing similar temporal expression pattern were created and overexpressed transcripts were selected from these groups.

### Detection of photosynthesis

*U. compressa* (25 mg of fresh tissue) was incubated in 2 mL of filtered seawater in the oxygraph chamber (Hansatech, model Oxygraph Plus, Norfolk, UK) and O_2_ production was measured for 10 min using a light intensity of 425 μmoles m^− 2^ s^− 1^.

### Quantification of carotenoids

The level of carotenoids was determined as described in [28] with modifications. *U. compressa* tissue (100 mg) was frozen in liquid nitrogen and homogenized in a mortar using a pestle. Ten mL of acetone (80%) were added, the mixture was shaken using a vortex for 10 s and incubated 15 min in ice. The mixture was centrifuged at 3000 rpm × 10 min at 4 °C and the supernatant was recovered. The absorbance was determined at 480 and 510 nm in a spectrophotometer (Agilent, model Cary 8454, CA, USA) and the level of carotenoids was determined using the formula:

$$ {\displaystyle \begin{array}{c}\mathrm{Carotenoids}\ \left(\mathrm{mg}\ {\mathrm{g}}^{-1}\right)=7.6\times {\mathrm{A}}_{480\ \mathrm{nm}}-1.49\times {\mathrm{A}}_{510\ \mathrm{nm}}\times \mathrm{DF}\times \mathrm{V}\times {\left(1\ \mathrm{cm}\times \mathrm{W}\times 1000\right)}^{-1}\\ {}\mathrm{V}=\mathrm{final}\ \mathrm{volume}\ \mathrm{of}\ \mathrm{chlorophyll}\ \mathrm{extract}\ \mathrm{in}\ 80\%\mathrm{acetone}\ \left(\mathrm{mL}\right)\\ {}\mathrm{W}=\mathrm{fresh}\ \mathrm{weight}\ \mathrm{of}\ \mathrm{leaf}\ \mathrm{tissue}\ \left(\mathrm{g}\right);\mathrm{DF}=\mathrm{Dilution}\ \mathrm{factor}\end{array}} $$

### Quantification of chlorophylls

The level of chlorophylls was determined as described in [29]. *U. compressa* (100 mg of FT) was frozen in liquid nitrogen and homogenized with 2 mL 80% acetone. The mixture was centrifuged at 3000 rpm for 10 min at 4 °C, the supernatant was recovered and diluted 10 times with 80% acetone. The absorbance was determined at 649 and 663 nm. The level of chlorophylls *a* and *b* was calculated according to the formula:

$$ \mathrm{Chlorophyll}\ a\ \left(\upmu \mathrm{g}\ {\mathrm{mL}}^{-1}\right)=12.7\times {\mathrm{A}}_{663\mathrm{nm}}-2.69\times {\mathrm{A}}_{645\mathrm{nm}} $$

$$ \mathrm{Chlorophyll}\ b\ \left(\upmu \mathrm{g}\ {\mathrm{mL}}^{-1}\right)=22.9\times {\mathrm{A}}_{645\mathrm{nm}}-4.68\times {\mathrm{A}}_{663\mathrm{nm}} $$

### Statistical analyses

Significant differences in oxygen production and carotenoid levels were calculated with one-way ANOVA at 95% confidence interval, followed by a Tukey’s multiple comparison post-test using the statistical software Prism 6 (GraphPad Software Inc., California, USA). Analyses were conducted as three independent replicates.

## Additional files

Additional file 1: **Figure S1.** Preprocessing of reads and transcriptome length distribution. (**a**) Percentage of preprocessed reads starting from total sequenced reads (X axis) per library (Y axis). “Filtered” shows the percentage of reads filtered out by the quality control (QC) process. “Bacteria” shows the percentage of bacterial reads (mapped against RefSeq’s bacteria database). “*U. compressa*” shows quality-controlled and decontaminated reads, which we used for downstream analysis. (**b**) Histogram showing the length distribution (bp; X axis) of the assembled transcripts (contigs; Y axis) of the *Ulva compressa* transcriptome. We assembled a total of 106,704 transcripts with an average length of 868 bp. (TIF 917 kb)

Additional file 2: **Figure S2.** Pie chart of the percentage of protein sequences associated with different molecular functions (**a**) and cellular component (**b**) obtained from the transcriptomic analyses performed in the marine alga *U. compressa* cultivated with 10 μM of copper for 0, 3, 6, 12 and 24 h. (TIF 2198 kb)

## Abbreviations

CD: Carotene desaturase
DT: Dry tissue
ER: Endoplasmic reticulum
FBP: Fructose 1,6 biphosphatase
FT: Fresh tissue
G3PDH: Glyceraldehyde 3 phosphate dehydrogenase
GGDS: Geranylgeranyl diphosphate synthase
LC: Lycopene β- cyclase
LHCI: Light harvesting complex I
LHCII: Light harvesting complex II
PD: Phytoene desaturase
PGK: Phosphoglycerate kinase
PHS: Phytoene synthase
PSI: Photosystem I
PSII: Photosystem II
R5PI: Ribose 5-phophate isomerase
RbcS: Small subunit of ribulose 1,5 biphosphate carboxylase/oxygenase (rubisco)
ROS: Reactive oxygen species
RP3E: Ribulose phosphate 3
SHBP: Seudoheptulose 1,7 biphosphatase phosphoribulokinase
TK: Transketolase
