# Supplementary figures and images for: Copper-induced increased expression of genes involved in photosynthesis, carotenoid synthesis and C assimilation in the marine alga Ulva compressa
Source: BMC Genomics. 2018 Nov 20;19:829. doi: 10.1186/s12864-018-5226-4 (PMC6245705; doi:10.1186/s12864-018-5226-4)

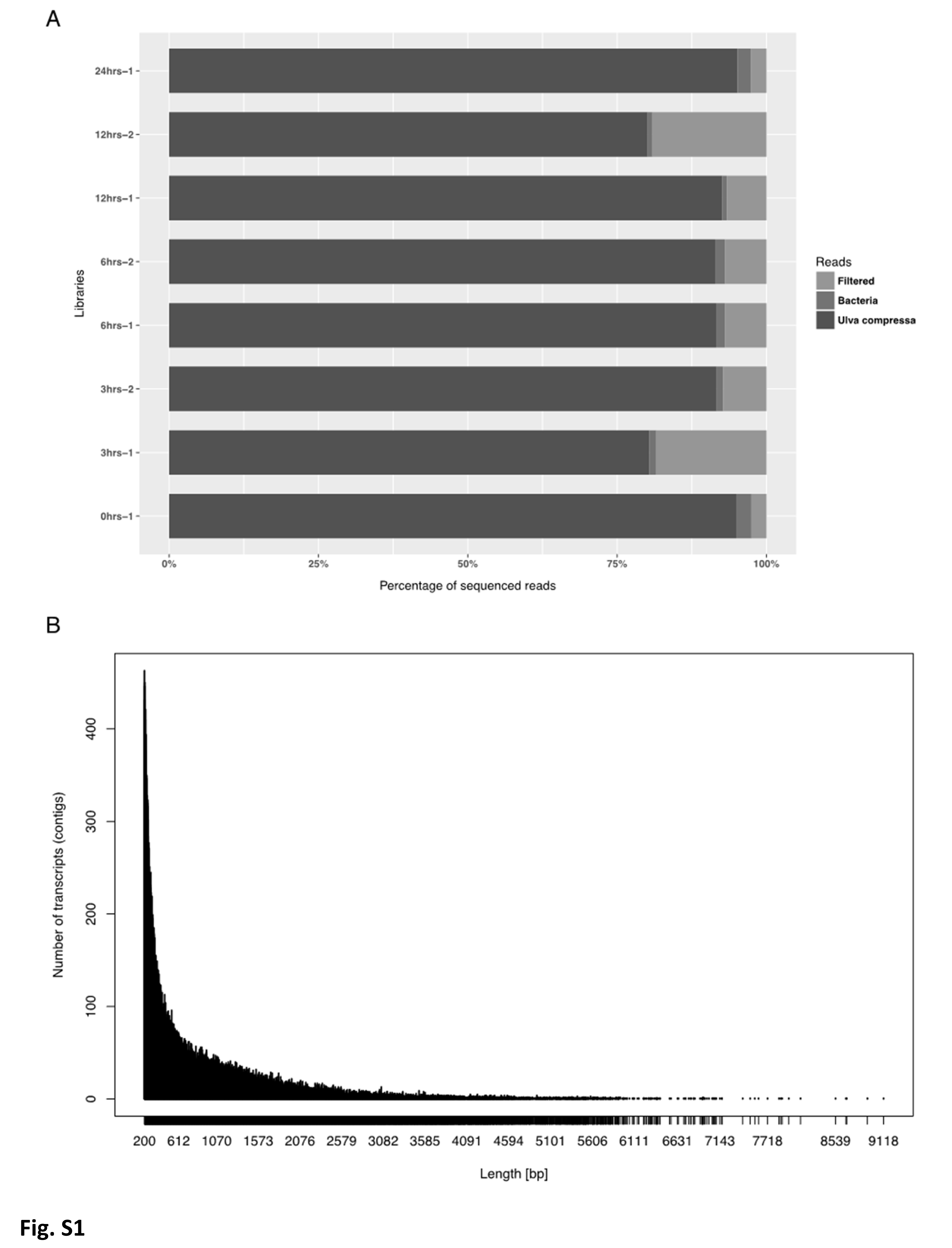

Supplement: Supplementary file 1 — Figure S1. Preprocessing of reads and transcriptome length distribution. (a) Percentage of preprocessed reads starting from total sequenced reads (X axis) per library (Y axis). “Filtered” shows the percentage of reads filtered out by the quality control (QC) process. “Bacteria” shows the percentage of bacterial reads (mapped against RefSeq’s bacteria database). “U. compressa” shows quality-controlled and decontaminated reads, which we used for downstream analysis. (b) Histogram showing the length distribution (bp; X axis) of the assembled transcripts (contigs; Y axis) of the Ulva compressa transcriptome. We assembled a total of 106,704 transcripts with an average length of 868 bp. (TIF 917 kb) [file 12864_2018_5226_MOESM1_ESM.tif]

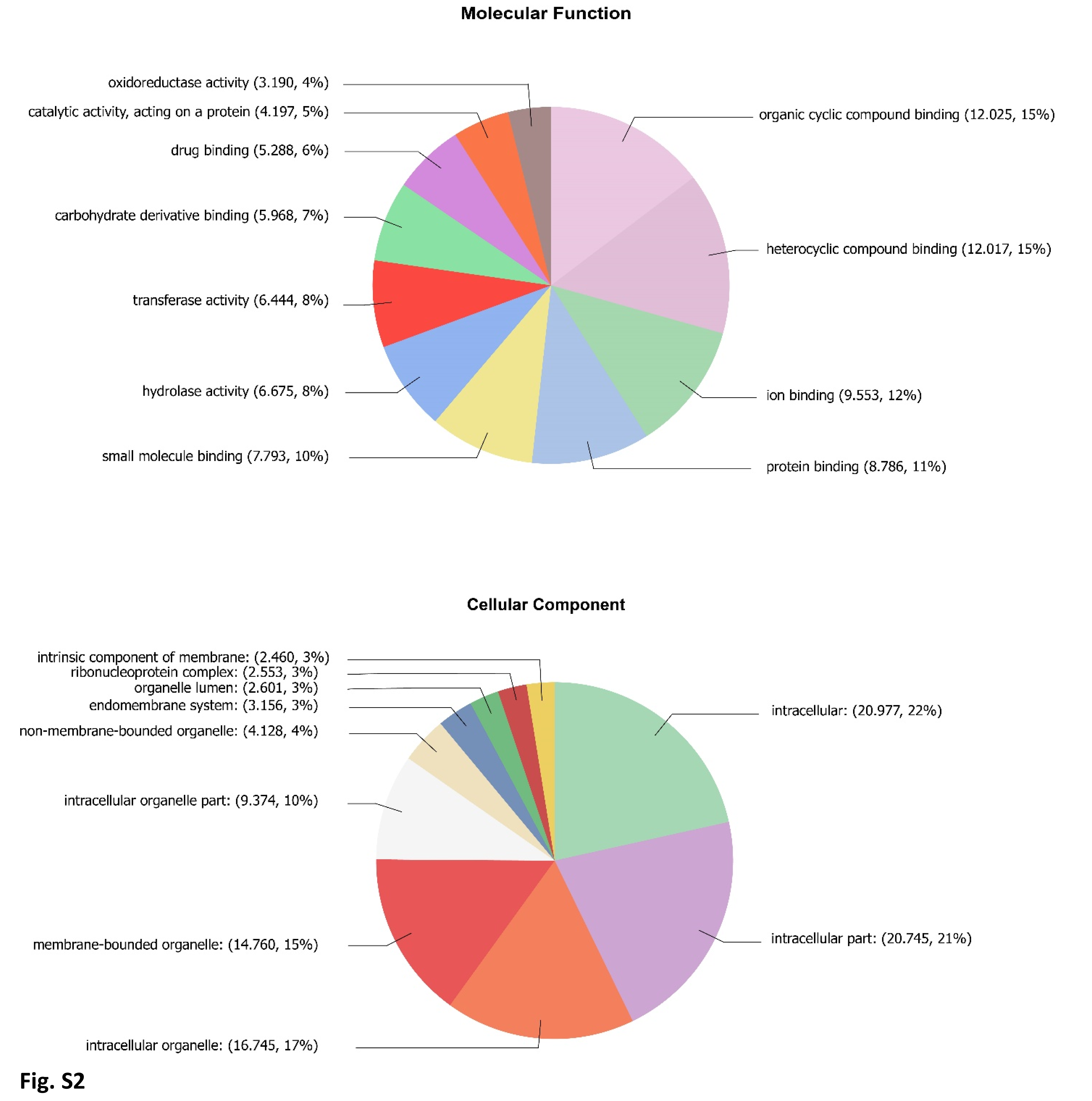

Supplement: Supplementary file 2 — Figure S2. Pie chart of the percentage of protein sequences associated with different molecular functions (a) and cellular component (b) obtained from the transcriptomic analyses performed in the marine alga U. compressa cultivated with 10 μM of copper for 0, 3, 6, 12 and 24 h. (TIF 2198 kb) [file 12864_2018_5226_MOESM2_ESM.tif]
